# Supplementary material for: Influence of emotions on clinical performance in acute care: A scoping review
Source: PLoS One. 2025 Aug 4;20(8):e0329445. doi: 10.1371/journal.pone.0329445 (PMC12321073; doi:10.1371/journal.pone.0329445)
Supplement: S1 Appendix — (DOCX) [file pone.0329445.s001.docx]

**Appendix -** Keywords used in the search strategy

**Medline**

1. exp Emotions/

2. Stress, Psychological/

3. exp Mental Fatigue/

4. exp Occupational Stress/

5. (emotion* or feeling* or regret* or affect* or anger* or angry or rage or rages or raging or anxiet* or anxious* or apath* or bereav* or grief or griev* or forgive* or happiness or happy or jealous* or hostil* or loneliness or lonely or sadness or pleasure or love or hope* or hate* or embarrass* or shame* or guilt* or panic* or fear* or euphoria* or distress* or disgust* or courage or boredom or bored or mood or moods or angst* or nervous* or hypervigilan* or mourning or brave* or contempt* or revulsion or unhapp* or gratification* or enjoy* or envy or envious or aggress* or afraid or scared or annoy* or furious or concern* or apprehens* or tense or worried or worry or exhilarat* or energetic or lively or vigorous or ashamed or bitter or bitterness or calm or peaceful or relax* or cheerful or overjoy* or joyful or happy or confiden* or daring or strong or confus* or deject* or uncertain* or sad or disappoint* or content* or settled or pleased or upset or dissatisf* or depressed or miserable or exhaust or exhausted or fatigue or sleepy or sluggish or frustrate or frustrated or frustration or irritable or irritability or irate or irritated or restless or restlessness or uneasy or uneasiness or unease or fulfil or fulfilment or satisfied or satisfy or satisfaction or delight or delightful or elated or ecstatic or excite or excited or excitement or determined or motivate or motivated or pressured or strained or stress or stressful or stressed or overwhelm or overwhelming or overwhelmed).ti,ab,kf.

6. or/1-5

7. Critical Care/

8. exp Ambulatory Care/

9. Hospitalization/

10. exp Emergency Service, Hospital/

11. Critical Illness/

12. ((acute or intensive or urgent or ambulatory or critical or emergency) adj2 care).ti,ab,kf.

13. (emergency adj2 (service* or department* or ward or wards or room or rooms or ward or wards or unit or units)).ti,ab,kf.

14. (trauma adj2 cent*).ti,ab,kf.

15. icu.ti,ab,kf.

16. or/7-15

17. exp Clinical Decision-Making/

18. Clinical Competence/

19. ((clinical or medical) adj2 (decision-making or reasoning*)).tw,kw.

20. (clinical adj2 judg*).tw,kw.

21. (clinical* adj2 competen*).tw,kw.

22. (clinical adj2 skill*).tw,kw.

23. (critical adj2 think*).tw,kw.

24. or/17-23

25. Physicians/

26. Hospitalists/

27. "Internship and Residency"/

28. (physician* or doctor* or residen* or "house staff" or hospitalist* or intern* or practitioner*).ti,ab,kf.

29. or/25-28

30. 6 and 16 and 24 and 29

**Embase**

1. emotion/ or exp anger/ or anhedonia/ or boredom/ or demoralization/ or disgust/ or euphoria/ or exp fear/ or frustration/ or exp grief/ or exp guilt/ or happiness/ or helplessness/ or hope/ or hopelessness/ or exp hostility/ or jealousy/ or mental irritation/ or mood/ or mood change/ or exp nervousness/ or pleasure/ or unhappiness/

2. mental fatigue/

3. mental stress/

4. job stress/

5. (emotion* or feeling* or regret* or affect* or anger* or angry or rage or rages or raging or anxiet* or anxious* or apath* or bereav* or grief or griev* or forgive* or happiness or happy or jealous* or hostil* or loneliness or lonely or sadness or pleasure or love or hope* or hate* or embarrass* or shame* or guilt* or panic* or fear* or euphoria* or distress* or disgust* or courage or boredom or bored or mood or moods or angst* or nervous* or hypervigilan* or mourning or brave* or contempt* or revulsion or unhapp* or gratification* or enjoy* or envy or envious or aggress* or afraid or scared or annoy* or furious or concern* or apprehens* or tense or worried or worry or exhilarat* or energetic or lively or vigorous or ashamed or bitter or bitterness or calm or peaceful or relax* or cheerful or overjoy* or joyful or happy or confiden* or daring or strong or confus* or deject* or uncertain* or sad or disappoint* or content* or settled or pleased or upset or dissatisf* or depressed or miserable or exhaust or exhausted or fatigue or sleepy or sluggish or frustrate or frustrated or frustration or irritable or irritability or irate or irritated or restless or restlessness or uneasy or uneasiness or unease or fulfil or fulfilment or satisfied or satisfy or satisfaction or delight or delightful or elated or ecstatic or excite or excited or excitement or determined or motivate or motivated or pressured or strained or stress or stressful or stressed or overwhelm or overwhelming or overwhelmed).ti,ab,kf.

6. or/1-5

7. intensive care/

8. ambulatory care/

9. hospitalization/

10. hospital emergency service/

11. critical illness/

12. exp intensive care unit/

13. ((acute or intensive or urgent or ambulatory or critical or emergency) adj2 care).ti,ab,kf.

14. (emergency adj2 (service* or department* or ward or wards or room or rooms or ward or wards or unit or units)).ti,ab,kf.

15. (trauma adj2 cent*).ti,ab,kf.

16. icu.ti,ab,kf.

17. or/7-16

18. clinical decision making/

19. clinical competence/

20. ((clinical or medical) adj2 (decision-making or reasoning*)).ti,ab,kf.

21. (clinical adj2 judg*).ti,ab,kf.

22. (clinical* adj2 competen*).ti,ab,kf.

23. (clinical adj2 skill*).ti,ab,kf.

24. (critical adj2 think*).ti,ab,kf.

25. or/18-24

26. physician/ or hospital physician/

27. resident/

28. (physician* or doctor* or residen* or "house staff" or hospitalist* or intern* or practitioner*).ti,ab,kf.

29. or/26-28

30. 6 and 17 and 25 and 29

**CENTRAL**

1. exp Emotions/

2. Stress, Psychological/

3. exp Mental Fatigue/

4. exp Occupational Stress/

5. (emotion* or feeling* or regret* or affect* or anger* or angry or rage or rages or raging or anxiet* or anxious* or apath* or bereav* or grief or griev* or forgive* or happiness or happy or jealous* or hostil* or loneliness or lonely or sadness or pleasure or love or hope* or hate* or embarrass* or shame* or guilt* or panic* or fear* or euphoria* or distress* or disgust* or courage or boredom or bored or mood or moods or angst* or nervous* or hypervigilan* or mourning or brave* or contempt* or revulsion or unhapp* or gratification* or enjoy* or envy or envious or aggress* or afraid or scared or annoy* or furious or concern* or apprehens* or tense or worried or worry or exhilarat* or energetic or lively or vigorous or ashamed or bitter or bitterness or calm or peaceful or relax* or cheerful or overjoy* or joyful or happy or confiden* or daring or strong or confus* or deject* or uncertain* or sad or disappoint* or content* or settled or pleased or upset or dissatisf* or depressed or miserable or exhaust or exhausted or fatigue or sleepy or sluggish or frustrate or frustrated or frustration or irritable or irritability or irate or irritated or restless or restlessness or uneasy or uneasiness or unease or fulfil or fulfilment or satisfied or satisfy or satisfaction or delight or delightful or elated or ecstatic or excite or excited or excitement or determined or motivate or motivated or pressured or strained or stress or stressful or stressed or overwhelm or overwhelming or overwhelmed).ti,ab,kf.

6. or/1-5

7. Critical Care/

8. exp Ambulatory Care/

9. Hospitalization/

10. exp Emergency Service, Hospital/

11. Critical Illness/

12. ((acute or intensive or urgent or ambulatory or critical or emergency) adj2 care).ti,ab,kf.

13. (emergency adj2 (service* or department* or ward or wards or room or rooms or ward or wards or unit or units)).ti,ab,kf.

14. (trauma adj2 cent*).ti,ab,kf.

15. icu.ti,ab,kf.

16. or/7-15

17. exp Clinical Decision-Making/

18. Clinical Competence/

19. ((clinical or medical) adj2 (decision-making or reasoning*)).tw,kw.

20. (clinical adj2 judg*).tw,kw.

21. (clinical* adj2 competen*).tw,kw.

22. (clinical adj2 skill*).tw,kw.

23. (critical adj2 think*).tw,kw.

24. or/17-23

25. Physicians/

26. Hospitalists/

27. "Internship and Residency"/

28. (physician* or doctor* or residen* or "house staff" or hospitalist* or intern* or practitioner*).ti,ab,kf.

29. or/25-28

30. 6 and 16 and 24 and 29

**APA PsycINFO**

1. exp emotions/

2. psychological stress/

3. occupational stress/

4. (emotion* or feeling* or regret* or affect* or anger* or angry or rage or rages or raging or anxiet* or anxious* or apath* or bereav* or grief or griev* or forgive* or happiness or happy or jealous* or hostil* or loneliness or lonely or sadness or pleasure or love or hope* or hate* or embarrass* or shame* or guilt* or panic* or fear* or euphoria* or distress* or disgust* or courage or boredom or bored or mood or moods or angst* or nervous* or hypervigilan* or mourning or brave* or contempt* or revulsion or unhapp* or gratification* or enjoy* or envy or envious or aggress* or afraid or scared or annoy* or furious or concern* or apprehens* or tense or worried or worry or exhilarat* or energetic or lively or vigorous or ashamed or bitter or bitterness or calm or peaceful or relax* or cheerful or overjoy* or joyful or happy or confiden* or daring or strong or confus* or deject* or uncertain* or sad or disappoint* or content* or settled or pleased or upset or dissatisf* or depressed or miserable or exhaust or exhausted or fatigue or sleepy or sluggish or frustrate or frustrated or frustration or irritable or irritability or irate or irritated or restless or restlessness or uneasy or uneasiness or unease or fulfil or fulfilment or satisfied or satisfy or satisfaction or delight or delightful or elated or ecstatic or excite or excited or excitement or determined or motivate or motivated or pressured or strained or stress or stressful or stressed or overwhelm or overwhelming or overwhelmed).ti,ab.

5. or/1-4

6. exp intensive care/

7. exp emergency services/

8. exp hospitalization/

9. ((acute or intensive or urgent or ambulatory or critical or emergency) adj2 care).ti,ab.

10. (emergency adj2 (service* or department* or ward or wards or room or rooms or ward or wards or unit or units)).ti,ab.

11. (trauma adj2 cent*).ti,ab.

12. icu.ti,ab.

13. or/6-12

14. "clinical judgment (not diagnosis)"/

15. decision making/

16. professional competence/

17. ((clinical or medical) adj2 (decision-making or reasoning*)).tw.

18. (clinical adj2 judg*).tw.

19. (clinical* adj2 competen*).tw.

20. (clinical adj2 skill*).tw.

21. (critical adj2 think*).tw.

22. or/14-21

23. exp physicians/

24. medical residency/

25. (physician* or doctor* or residen* or "house staff" or hospitalist* or intern* or practitioner*).ti,ab.

26. or/23-25

27. 5 and 13 and 22 and 26

**ERIC**

1. exp emotions/

2. Psychological Patterns/

3. (emotion* or feeling* or regret* or affect* or anger* or angry or rage or rages or raging or anxiet* or anxious* or apath* or bereav* or grief or griev* or forgive* or happiness or happy or jealous* or hostil* or loneliness or lonely or sadness or pleasure or love or hope* or hate* or embarrass* or shame* or guilt* or panic* or fear* or euphoria* or distress* or disgust* or courage or boredom or bored or mood or moods or angst* or nervous* or hypervigilan* or mourning or brave* or contempt* or revulsion or unhapp* or gratification* or enjoy* or envy or envious or aggress* or afraid or scared or annoy* or furious or concern* or apprehens* or tense or worried or worry or exhilarat* or energetic or lively or vigorous or ashamed or bitter or bitterness or calm or peaceful or relax* or cheerful or overjoy* or joyful or happy or confiden* or daring or strong or confus* or deject* or uncertain* or sad or disappoint* or content* or settled or pleased or upset or dissatisf* or depressed or miserable or exhaust or exhausted or fatigue or sleepy or sluggish or frustrate or frustrated or frustration or irritable or irritability or irate or irritated or restless or restlessness or uneasy or uneasiness or unease or fulfil or fulfilment or satisfied or satisfy or satisfaction or delight or delightful or elated or ecstatic or excite or excited or excitement or determined or motivate or motivated or pressured or strained or stress or stressful or stressed or overwhelm or overwhelming or overwhelmed).ti,ab.

4. or/1-3

5. ((acute or intensive or urgent or ambulatory or critical or emergency) adj2 care).ti,ab.

6. (emergency adj2 (service* or department* or ward or wards or room or rooms or ward or wards or unit or units)).ti,ab.

7. (trauma adj2 cent*).ti,ab.

8. icu.ti,ab.

9. or/5-8

10. decision making/

11. clinical experience/

12. ((clinical or medical) adj2 (decision-making or reasoning*)).tw.

13. (clinical adj2 judg*).tw.

14. (clinical* adj2 competen*).tw.

15. (clinical adj2 skill*).tw.

16. (critical adj2 think*).tw.

17. or/10-16

18. exp physicians/

19. (physician* or doctor* or residen* or "house staff" or hospitalist* or intern* or practitioner*).ti,ab.

20. or/18-19

21. 4 and 9 and 17 and 20

**CINAHL**

**Search History**

| \| **#** \| **Query** \| \| --- \| --- \| \| S21 \| S5 AND S13 AND S16 AND S20 \| \| S20 \| S17 OR S18 OR S19 \| \| S19 \| TI ( (physician* or doctor* or residen* or "house staff" or hospitalist* or intern* or practitioner*) ) OR AB ( (physician* or doctor* or residen* or "house staff" or hospitalist* or intern* or practitioner*) ) \| \| S18 \| (MH "Internship and Residency+") \| \| S17 \| (MH "Physicians") OR (MH "Hospitalists") OR (MH "Physicians, Emergency") \| \| S16 \| S14 OR S15 \| \| S15 \| TI ( ((clinical or medical) N2 (decision-making or reasoning* or judg* or competen* or skill* or think*)) ) OR AB ( ((clinical or medical) N2 (decision-making or reasoning* or judg* or competen* or skill* or think*)) ) \| \| S14 \| (MH "Decision Making, Clinical") OR (MH "Clinical Reasoning") OR (MH "Clinical Prediction Rules") \| \| S13 \| S6 OR S7 OR S8 OR S9 OR S10 OR S11 OR S12 \| \| S12 \| TI (trauma N2 cent*) OR AB (trauma N2 cent*) \| \| S11 \| TI ( (emergency N2 (service* or department* or ward or wards or room or rooms or ward or wards or unit or units)) ) OR AB ( (emergency N2 (service* or department* or ward or wards or room or rooms or ward or wards or unit or units)) ) \| \| S10 \| TI ( ((acute or intensive or urgent or ambulatory or critical or emergency) N2 care) ) OR AU ( ((acute or intensive or urgent or ambulatory or critical or emergency) N2 care) ) \| \| S9 \| (MH "Critical Illness") \| \| S8 \| (MH "Emergency Service") OR (MH "Trauma Centers") \| \| S7 \| (MH "Hospitalization") \| \| S6 \| (MH "Critical Care") OR (MH "Rapid Response (Emergency Care)") OR (MH "Ambulatory Care") \| \| S5 \| S1 OR S2 OR S3 OR S4 \| \| S4 \| TI ( (emotion* or feeling* or regret* or affect* or anger* or angry or rage or rages or raging or anxiet* or anxious* or apath* or bereav* or grief or griev* or forgive* or happiness or happy or jealous* or hostil* or loneliness or lonely or sadness or pleasure or love or hope* or hate* or embarrass* or shame* or guilt* or panic* or fear* or euphoria* or distress* or disgust* or courage or boredom or bored or mood or moods or angst* or nervous* or hypervigilan* or mourning or brave* or contempt* or revulsion or unhapp* or gratification* or enjoy* or envy or envious or aggress* or afraid or scared or annoy* or furious or concern* or apprehens* or tense or worried or worry or exhilarat* or energetic or lively or vigorous or ashamed or bitter or bitterness or calm or peaceful or relax* or cheerful or overjoy* or joyful or happy or confiden* or daring or strong or confus* or deject* or uncertain* or sad or disappoint* or content* or settled or pleased or upset or dissatisf* or depressed or miserable or exhaust or exhausted or fatigue or sleepy or sluggish or frustrate or frustrated or frustration or irritable or irritability or irate or irritated or restless or restlessness or uneasy or uneasiness or unease or fulfil or fulfilment or satisfied or satisfy or satisfaction or delight or delightful or elated or ecstatic or excite or excited or excitement or determined or motivate or motivated or pressured or strained or stress or stressful or stressed or overwhelm or overwhelming or overwhelmed) ) OR AB ( (emotion* or feeling* or regret* or affect* or anger* or angry or rage or rages or raging or anxiet* or anxious* or apath* or bereav* or grief or griev* or forgive* or happiness or happy or jealous* or hostil* or loneliness or lonely or sadness or pleasure or love or hope* or hate* or embarrass* or shame* or guilt* or panic* or fear* or euphoria* or distress* or disgust* or courage or boredom or bored or mood or moods or angst* or nervous* or hypervigilan* or mourning or brave* or contempt* or revulsion or unhapp* or gratification* or enjoy* or envy or envious or aggress* or afraid or scared or annoy* or furious or concern* or apprehens* or tense or worried or worry or exhilarat* or energetic or lively or vigorous or ashamed or bitter or bitterness or calm or peaceful or relax* or cheerful or overjoy* or joyful or happy or confiden* or daring or strong or confus* or deject* or uncertain* or sad or disappoint* or content* or settled or pleased or upset or dissatisf* or depressed or miserable or exhaust or exhausted or fatigue or sleepy or sluggish or frustrate or frustrated or frustration or irritable or irritability or irate or irritated or restless or restlessness or uneasy or uneasiness or unease or fulfil or fulfilment or satisfied or satisfy or satisfaction or delight or delightful or elated or ecstatic or excite or excited or excitement or determined or motivate or motivated or pressured or strained or stress or stressful or stressed or overwhelm or overwhelming or overwhelmed) ) \| \| S3 \| (MH "Mental Fatigue") \| \| S2 \| (MH "Stress, Psychological") OR (MH "Stress, Occupational") \| \| S1 \| (MH "Emotions+") \| |
| --- | --- | --- | --- | --- | --- | --- | --- | --- | --- | --- | --- | --- | --- | --- | --- | --- | --- | --- | --- | --- | --- | --- | --- | --- | --- | --- | --- | --- | --- | --- | --- | --- | --- | --- | --- | --- | --- | --- | --- | --- | --- | --- | --- | --- |
