# Supplementary material for: Influence of emotions on clinical performance in acute care: A scoping review
Source: PLoS One. 2025 Aug 4;20(8):e0329445. doi: 10.1371/journal.pone.0329445 (PMC12321073; doi:10.1371/journal.pone.0329445)
Supplement: Attachment — Submitted filename: comments.docx [file pone.0329445.s003.docx]

This manuscript provides a well-executed scoping review examining how emotions influence clinical performance in acute care settings. It addresses a significant and understudied issue in healthcare—how affective states (both positive and negative) shape clinical decision-making, skill performance, and provider behavior. The manuscript follows appropriate scoping review methodology, reports data clearly, and draws meaningful implications for clinical training and future research.

**Comments:**

The paper sometimes uses terms like “integral” and “incidental” emotions without early definition. These should be clearly defined in the introduction or methods for a broader audience. “Negative emotions” is used as a catch-all term—more nuanced language (e.g., anger, fear, sadness, stress) could improve interpretability in certain sections. Add a table summarizing emotional constructs across studies (e.g., specific emotions examined, definitions used, measurement methods).

While scoping reviews don't typically require a formal quality appraisal, readers would benefit from a brief comment on the general methodological quality of the included studies (e.g., experimental vs. observational vs. qualitative). Include a short paragraph noting the predominant study designs and potential limitations in evidence quality.

Most of the analysis focuses on negative emotional states. While this reflects the literature, the potential benefits of positive emotions (e.g., joy, compassion, gratitude) in clinical performance are underexplored in the discussion. Strengthen discussion on how positive emotions could be leveraged in training or decision-making.

The section on end-of-life care lacks clear connections between emotional states and specific performance outcomes. Acknowledge this more explicitly in the discussion as a gap in the literature and a target for future research.
