# Supplementary material for: Influence of emotions on clinical performance in acute care: A scoping review
Source: PLoS One. 2025 Aug 4;20(8):e0329445. doi: 10.1371/journal.pone.0329445 (PMC12321073; doi:10.1371/journal.pone.0329445)
Supplement: Attachment — Submitted filename: Response to Reviewers.docx [file pone.0329445.s005.docx]

**Reviewer #1 Comments:**

1. **“The paper sometimes uses terms like “integral” and “incidental” emotions without early definition. These should be clearly defined in the introduction or methods for a broader audience.”**

Response: This is a good point, and we previously decided to clarify these terms in the discussion, as we did not anticipate this distinction to come up with the results. We added a section within the Introduction to define integral and incidental emotions and provide examples of how they affect clinical performance differently within the current literature (the paragraph starting line 44. We also provided evidence from LeBlanc et al. that integral emotions can direct attention to a clinical task while incidental emotions can direct attention away.

2. **“Negative emotions” is used as a catch-all term—more nuanced language (e.g., anger, fear, sadness, stress) could improve interpretability in certain sections. Add a table summarizing emotional constructs across studies (e.g., specific emotions examined, definitions used, measurement methods).”**

Response: Thank you for this comment. Rather than add an entirely new table, we added a column to Table 2 to summarize the emotional constructs across the studies, and the measurement of these constructs (where applicable). We also altered text throughout the manuscript to be more specific, referencing specific emotional states whenever possible. Admittedly, part of the challenge we had is that most studies did not define emotions, or the definitions were not applicable. As such, we also elaborated on the lack of clear definitions of emotional constructs in our Discussion section (lines 472-477).

3. **“While scoping reviews don't typically require a formal quality appraisal, readers would benefit from a brief comment on the general methodological quality of the included studies (e.g., experimental vs. observational vs. qualitative). Include a short paragraph noting the predominant study designs and potential limitations in evidence quality.”**

Response: Several paragraphs discussing the most predominant methodologies and their strengths and limitations were added just prior to the Limitations section (paragraph starting Line 459). In addition to elaborating on the strengths and limitations of the different types of methodologies used by the studies in our review, we also noted that there was a lack of theoretical or conceptual clarity regarding emotional constructs (see Comment 2, above).

4. **“Most of the analysis focuses on negative emotional states. While this reflects the literature, the potential benefits of positive emotions (e.g., joy, compassion, gratitude) in clinical performance are underexplored in the discussion. Strengthen discussion on how positive emotions could be leveraged in training or decision-making.”**

Response: A section on the effects of positive emotions on patient outcomes was added in the paragraph that discusses the practical implications of our findings (starting Line 439). We stated that “while most studies have focused on negative emotional states, emerging literature suggests that positive emotions may also enhance clinical performance” and provided two examples. We further stated that “these findings highlight the potential value of further exploring the role of positive emotions in clinical performance and identifying strategies to leverage these emotional states to improve patient outcomes”.

5. **“The section on end-of-life care lacks clear connections between emotional states and specific performance outcomes. Acknowledge this more explicitly in the discussion as a gap in the literature and a target for future research.”**

Response: The description on this research gap was revised in the paragraph starting Line 376. The wording was changed to more explicitly identify this research gap and the need to address it in future research. We stated that “the studies reviewed did not identify or define the types of emotions experienced by healthcare professionals (e.g., stress, guilt, compassion, or joy), nor did they specify the clinical decisions influenced by these emotional states.” and “future research should aim to identify the specific emotions elicited by such conflicts and examine how these emotional experiences influence care decisions and clinical performance in end-of-life settings.”

**Reviewer #2 Comment:**

1. **“However, for the introduction part, it is suggested that the author to add more explanation on clinical performance and acute care setting.”**

Response: This comment has been addressed in the paragraph starting Line 62. First, we provided elaboration on what we mean by “acute care” and “clinical performance”. We added a section that provided more detail on the time-sensitivity of cute care, using the “door-to-needle time” of acute myocardial infarction as an example. Additionally, we stated that “healthcare professionals report a wide range of emotions being present in the acute care setting” and “the acute care setting consists of interdisciplinary healthcare professionals, and the emotion skills training of these professional are heterogenous”. We believe these additional details help contextualize why the acute care setting is uniquely suited to assess the effect of emotions on clinical performance across different healthcare specialties.
